# A Novel Automated Mass Digitisation Workflow for Natural History Microscope Slides

**DOI:** 10.3897/BDJ.7.e32342

**Published:** 2019-03-01

**Authors:** E Louise Allan, Laurence Livermore, Benjamin W Price, Olha Shchedrina, Vincent S Smith

**Affiliations:** 1 Natural History Museum, London, United Kingdom Natural History Museum London United Kingdom

**Keywords:** mass digitisation, automation, microscope slides, Phthiraptera, Data Matrix barcodes, natural history collections

## Abstract

The Natural History Museum, London (NHM) has embarked on an ambitious programme to digitise its collections. One aim of the programme has been to improve the workflows and infrastructure needed to support high-throughput digitisation and create comprehensive digital inventories of large scientific collections. This paper presents the workflow developed to digitise the entire Phthiraptera (parasitic lice) microscope slide collection (70,663 slides). Here we describe a novel process of semi-automated mass digitisation using both temporary and permanent barcode labels applied before and during slide imaging. By using a series of barcodes encoding information associated with each slide (i.e. unique identifier, location in the collection and taxonomic name), we can run a series of automated processes, including file renaming, image processing and bulk import into the NHM’s collection management system. We provide data on the comparative efficiency of these processes, illustrating how simple activities, like automated file renaming, reduces image post-processing time, minimises human error and can be applied across multiple collection types.

## Introduction

Digital surrogates of natural history specimens, comprising a combination of specimen data and images, are creating new audiences and new research opportunities for natural history collections (Drew et al. 2017, Decker et al. 2018). These digital copies not only provide an unprecedented level of access to data that has hitherto been restricted to those with privileged physical access to the specimens, but the scope, scale and speed with which new digital records can be acquired, are transforming our understanding of the natural world. It is now possible to construct datasets with millions of records, with global coverage, drawing on a mix of historical and contemporary observations of species to address questions such as why the natural world is changing, how humans are influencing this change and what we might do to minimise this loss (Purvis et al. 2018). The greatest barrier to this digital transformation is the relatively low proportion of specimens, especially entomological specimens, that have digital data (Sikes et al. 2016). In 2014, the Natural History Museum, London (NHM) embarked on an ambitious Digital Collections Programme (DCP) to digitise its collections, estimated to comprise 80 million specimens. One of the aims of the DCP is to develop new digitisation workflows to speed up the efficiency of mass digitisation.

**Figure 1.**
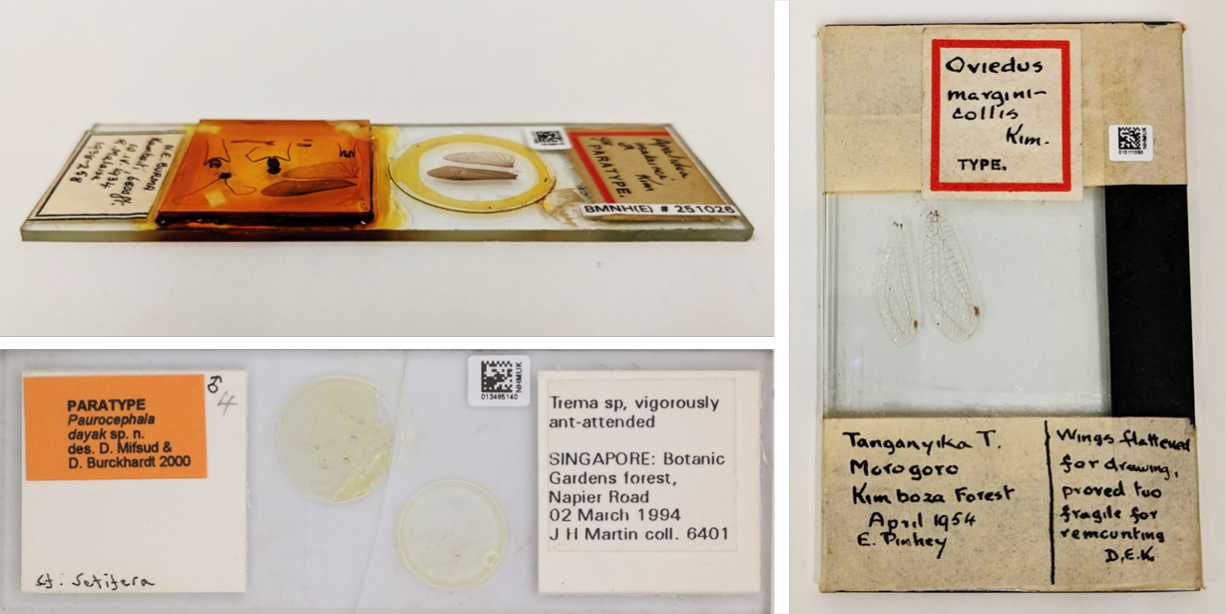
Examples of natural history microscope slides that are damaged or non-standard in size and mountant thickness.

We estimate that the NHM holds 2.4 million microscope slides in its collection. These slides are distributed across diverse curatorial groups (e.g. botany, entomology, mineralogy, palaeontology and zoology), with each group having its own distinct slide preparation technique and standards. While automated slide digitisation systems, designed for higher resolution imaging, have existed for over a decade, these have been confined to medical slides with no other large-scale digitisation projects of natural history slides known to us (Rojo et al. 2006, Weinstein et al. 2009, Dietrich et al. 2012). While there have been several pilot projects that have used specially modified histology slide scanners adapted for natural history specimens, they cannot accommodate damaged slides or slides with non-standard thickness or length (Musson et al. 2015, Summerfield et al. 2019) - issues that can be frequent in natural history collections (Fig. 1).

For natural history slide collections, the data on the labels is as important as the slide mounted material / specimen(s). While many of these automated scanners are able to capture a low resolution overview image of the slide, these images tend to be poorly lit, which could impact automated label extraction through optical character recognition. Furthermore, slide holders may partially obscure label data, while labels on the reverse side of the slide cannot currently be captured.

**Figure 2.**
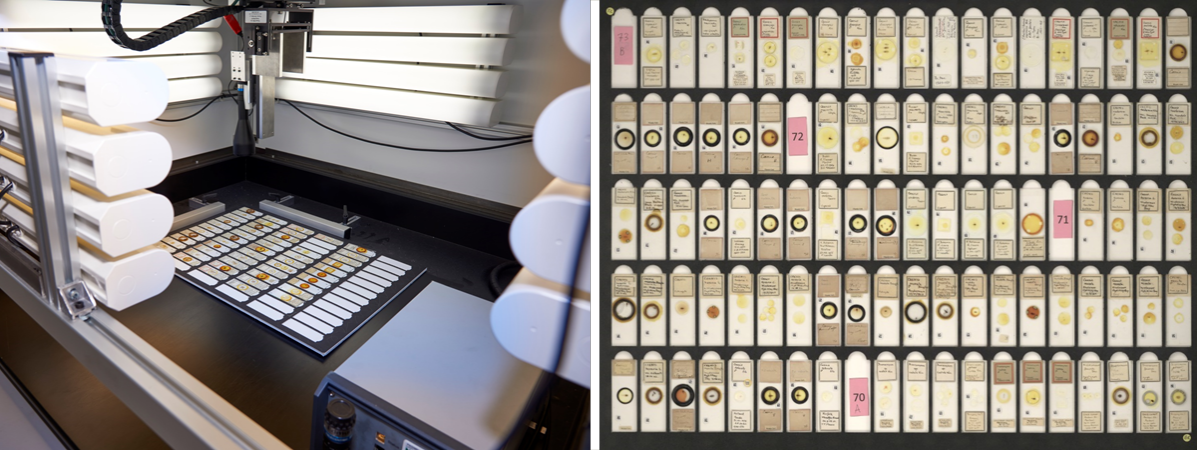
For the previous slide digitisation pilot batches of up to 100 slides were placed in a template and imaged using the SatScan^TM^.

In 2015, the Museum’s DCP ran a pilot project for mass digitisation of microscope slides using a multi-slide imaging template and downstream image segmentation (Summerfield et al. 2019), similar to that run at Naturalis, Leiden (Heerlien et al. 2015). This pilot project utilised a volunteer workforce of 45 people, in teams of 3 - 7 people per day, to scan ~100,000 microscope slides over 10 months using the SatScan^TM^ (Smartdrive Ltd.) and industrial approaches as described by Blagoderov et al. (2012). Batches of up to 100 slides were place in a template and multiple ‘tile’ images were captured (Fig. 2). Software then stitched these images together to create the final multi-slide image, which was subsequently processed with in-house open source software (*Inselect*: Hudson et al. 2015). Using *Inselect*, each slide was segmented out and tagged with the specimen’s metadata (e.g. taxonomy, collection location) using drop-down lists. The processing of images consisted of both manual and semi-automated steps, requiring substantial post-processing time and resulting in additional quality control steps to check for tagging errors.

In order to increase the efficiency and accuracy of digitisation workflows, more automated processes are needed. In 2017, the DCP began the digitisation of the microscope slide collection of Phthiraptera (~70,000 vertically stored slides). This scientific and culturally important collection is one of the largest of its kind in the world, containing a significant number of previously unidentified species, as well as a vast dataset of host-parasite associations that can only be exploited through transcription of the label data. The aim of the Phthiraptera slide digitisation project was to improve the existing workflow through pre-digitisation preparation of a collection, simplified slide imaging and automated extraction / post-processing of metadata.

This paper addresses four of the five digitisation task clusters outlined by Nelson et al. (2012):

1. pre-digitisation curation and staging;
2. specimen image capture;
3. specimen image processing;
4. electronic data capture.

In this project, we omitted the final task cluster (georeferencing of specimen data), as our aim was to develop a high-throughput inventory record of the collection and rapid digitisation workflow. Georeferencing remains one of the most challenging and slowest components of digitisation and is less relevant to the digitisation of parasitic lice, since their range is largely circumscribed by the distribution of the host bird or mammal.

## General Description

Mass digitisation of specimens requires the creation of an ‘inventory’ specimen record for each object within the Museum’s collection management system (CMS), EMu (© Axiell), consisting of three essential pieces of information: 1) a unique identifier (UID) catalogue number; 2) the current physical location of the specimen in the collection (e.g. cabinet and drawer); and 3) the taxonomic name of the specimen, as currently assigned in the collection.

The Museum’s CMS uses primary keys (numerical values) to uniquely identify location and taxon values, which eliminates the potential for erroneously matching synonyms. Two scripts were developed by Axiell during the 2015 slide digitisation pilot, for the bulk ingestion of specimen images and their associated data into the CMS:

1. **Specimen record creation**: This script takes individual images with metadata encoded in the filename and creates a specimen record with appropriate links to the relevant database tables providing the specimen’s UID, location and taxonomy. In order to create a specimen record using this script, the corresponding location and taxon information are exported as primary keys from the CMS. Example format of encoded metadata: “UIDBarcode_LocationPrimaryKey_TaxonPrimaryKey.jpg”. *Note*: to ensure duplicate specimen records are not created, the script checks the CMS for a pre-existing record with that UID (if a record already exists, the image file will be attached to the existing record; if no record exists, the script will create a new specimen record).
2. **Record attachment**: This script takes individual images and attaches them to a pre-existing record by matching the UID in the filename with an existing record in the CMS. Example format of encoded metadata: “UIDBarcode_suffix.jpg”. A suffix is used to ensure unique filenames and can be changed depending on the nature of the image i.e. labels only, labels on reverse side of a slide, paper envelopes, high resolution specimen images etc.

The use of primary keys ensures a 1:1 match in the CMS; however manual renaming of files to numerical values is prone to transcription error. Furthermore, numerical values for location and taxon cannot be easily verified before import into the CMS.

The previous slide digitisation pilot (Summerfield et al. 2019) developed a workaround for the manual renaming of files to primary key values through the use of *Inselect*, where human readable values, in a drop-down list, were automatically associated with their corresponding primary keys. The slides were segmented and tagged with their location and taxon using these drop-down lists. The segmented images were exported and renamed with the primary key values in the required format for the specimen record creation script.

This approach not only required substantial post-processing time, but also had many limitations. For example, the slides had to be manually tagged using pre-populated drop-down lists and, if there were discrepancies between the list and the physical collection i.e. a missing location and/or taxon, this would halt the tagging process and increase the post-processing time for each slide. Furthermore, as manual tagging of specimen images can also be prone to human error, additional verification steps were needed.

Scripts were also developed in-house to 1) facilitate transfer of image files within a staging area and 2) to ensure the clear down of the original image files from the imaging computer once ingested into the CMS.

## Project Description and Workflow

Our Phthiraptera slide digitisation project was designed to reuse the existing bulk ingest scripts; however, additional automated post-processing steps were developed to increase the efficiency of specimen digitisation and reduce the potential for human error. This was accomplished via automated file renaming of the images by incorporating additional barcodes in each image that encoded location and taxon primary key values associated with each specimen. This process had a dramatic impact in improving the efficiency of the digitisation process.

Another key difference between the Phthiraptera slide project and the previous pilot was the change to imaging individual slides rather than batches of slides in templates. This significantly reduced the number of steps required to create a single slide image as well as the amount of specimen handling.

**Figure 3.**
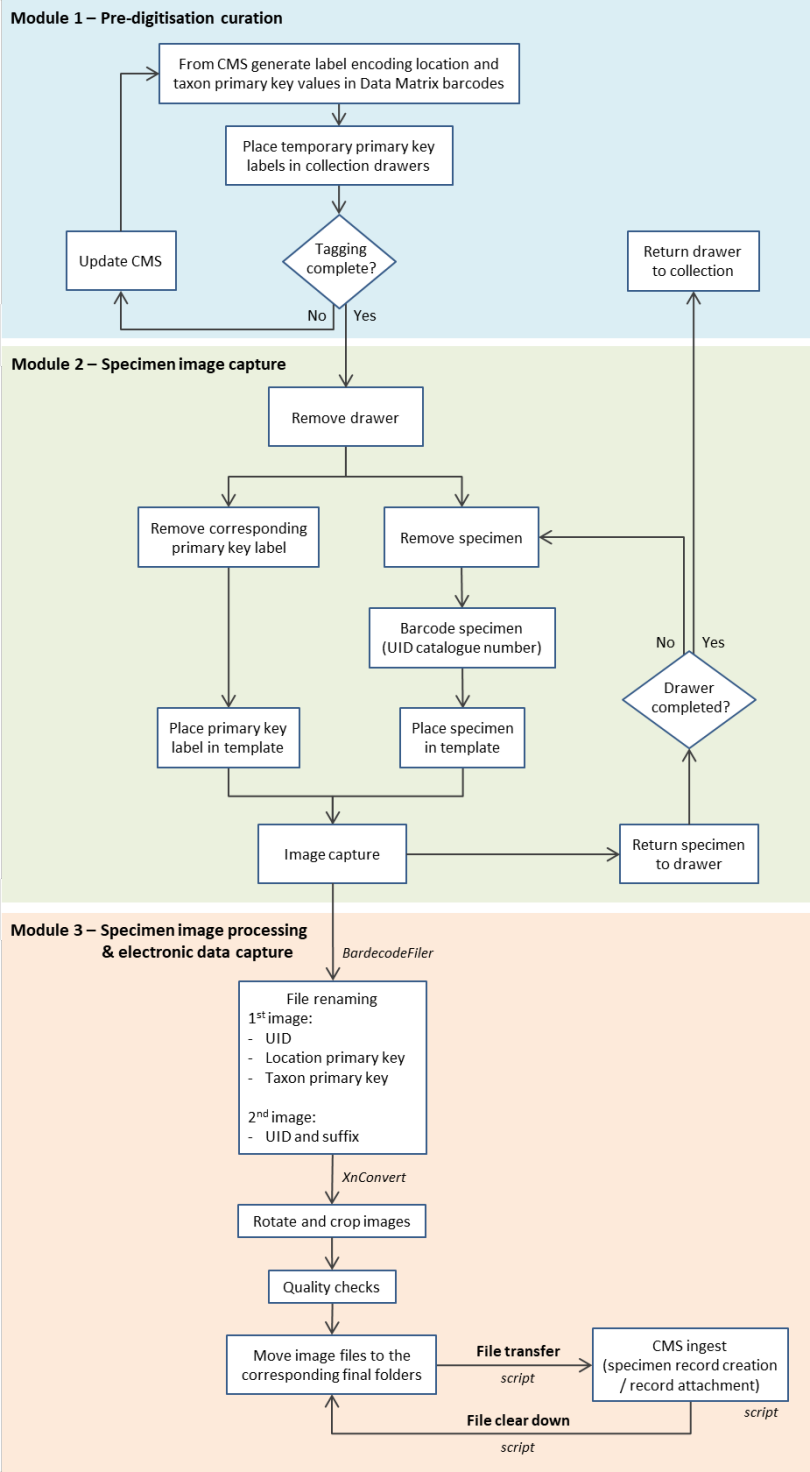
High throughput slide digitisation workflow using multiple barcodes to encode metadata to enable automated file renaming and bulk ingestion into a collection management system (CMS).

The workflow for whole slide digitisation is summarised below and consists of three modules: 1) pre-digitisation curation, 2) specimen image capture and 3) specimen image processing and electronic data capture (Fig. 3). It is also available as a step by step protocol (Allan et al. 2018a): https://dx.doi.org/10.17504/protocols.io.vmte46n

### Module 1: Pre-digitisation curation

**Figure 4.**
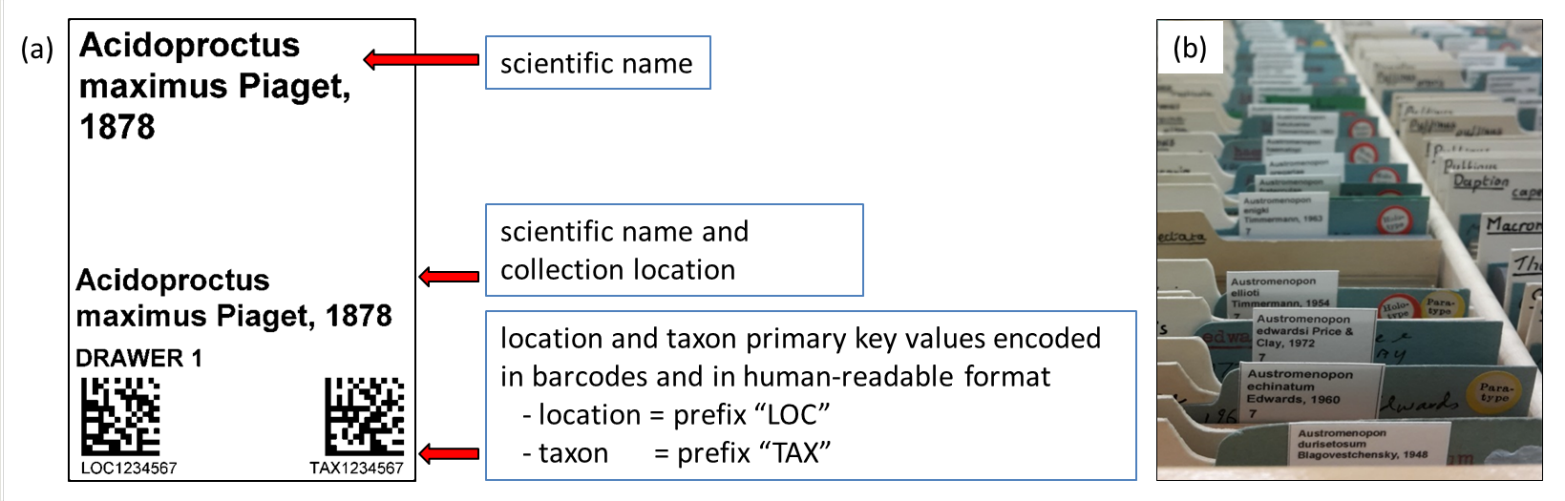
(a) An example of the temporary location and taxon primary key label, encoding these values in two machine-readable Data Matrix barcodes. (b) Temporary location and taxon labels inserted into a slide collection. This label was designed for vertical slide collections but can be adapted for other collection types.

1. Temporary location and taxon primary key labels were printed directly from the Museum's CMS to ensure a 1:1 match.

- These labels consisted of human-readable information as well as two machine-readable barcodes (Data Matrix ECC 200) that encoded the location and taxon primary key values (example shown in Fig. 4a).
- The location and taxon primary key values can either be produced as a single label using the index lot association in the catalogue module or as separate labels using the location and taxon modules.

2. The temporary labels were physically inserted into the collection prior to digitisation with curatorial overview (Fig. 4b). If specimens in the collection were missing a label, or information was incorrect, the CMS was updated by the curator.

3. Once pre-digitisation curation was completed, the drawers were moved to the digitisation station where they were temporarily stored while being digitised.

### Module 2: Specimen image capture

**Figure 5.**
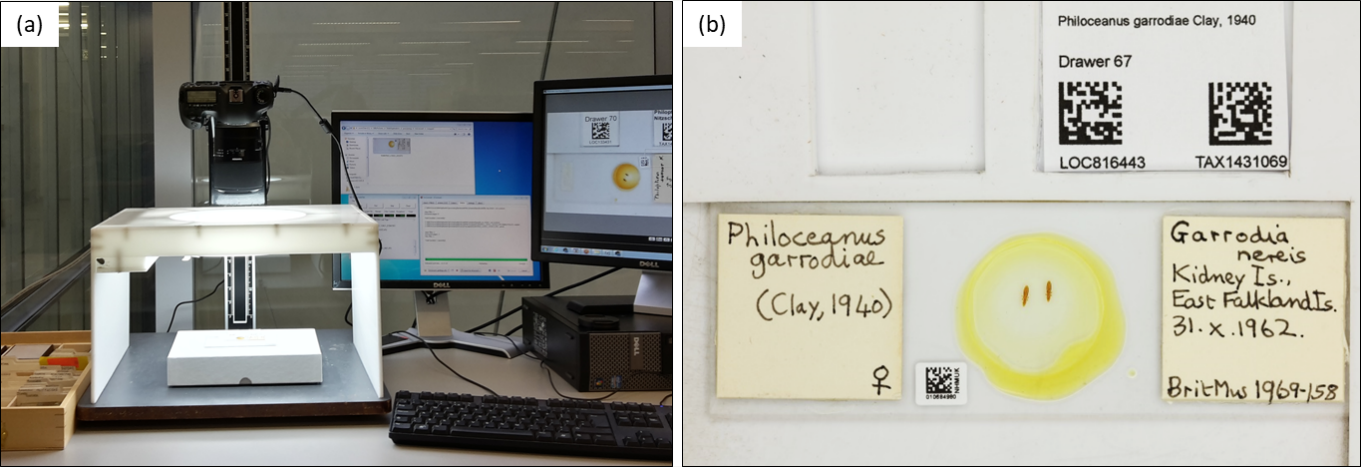
(a) Imaging setup consisting of a vertically mounted DSLR camera, a custom-built lightbox and a slide imaging template fixed in place. (b) Slide imaging template consisting of a raised ‘L-shaped’ edge, where the slide is positioned and a grooved area where the temporary location and taxon primary key label is placed within the field of view.

1. Before imaging, every slide was given a conservation grade self-adhesive UID barcode label (Data Matrix), which was attached to the glass, if possible, on the upper side of the slide using forceps.

- These UIDs (catalogue numbers) were generated from the Museum’s CMS to ensure unique values and were printed in both a human- and machine-readable format. For consistency and ease of application, the barcode labels are 5 x 6 mm, such that they can be applied without obscuring other label data, fit a range of collection types including slides with limited space and can be reliably read by barcode reading software. The barcode labels, supplied on rolled sheets, were printed by an external supplier on 8100 white polyester and use a permanent solvent acrylic adhesive.
- The UID can be used to associate additional images or data with a specimen record either by using the record attachment script or data ingestion processes. Some specimens already had an attached UID barcode label and specimen record in the CMS, as they were either digitised as part of a previous project or when sent on loan.

2. The slide, with its UID barcode, was then placed on to the imaging template along with the corresponding location and taxon primary key label.

- The template was fixed in place within a custom-built lightbox and positioned beneath a vertically mounted camera (Fig. 5a).
- The template was made from durable white plastic and had a raised ‘L-shaped’ edge to ensure that the microscope slides could be consistently and easily positioned in the same location to enable automated image processing (Fig. 5b). The ‘L-shaped’ edge contained a grooved area to place the primary key label so that it was positioned above the slide, but within the field of view.

3. Images were captured using a Canon EOS 5DS R and a 90 mm Tamron lens using Canon EOS Utility v.3.9.0 and saved to a hot folder (folder name: ‘input’).

- The camera mode was set to manual with the aperture set between f 1/5.6 and 1/7.1; ISO 200 and shutter speed 1/80 sec. The light source consisted of a custom-built lightbox with a 32 W Circline VLR Full Spectrum Vita-Lite 5500 K fluorescent ring bulb.

4. If labels were present on the reverse side of the slide, it was turned over and an additional image was captured. Likewise, if the slide was housed in a paper envelope with additional information, the envelope was placed in the template and an additional image was captured.

### Module 3: Specimen image processing and electronic data capture

**Figure 6.**
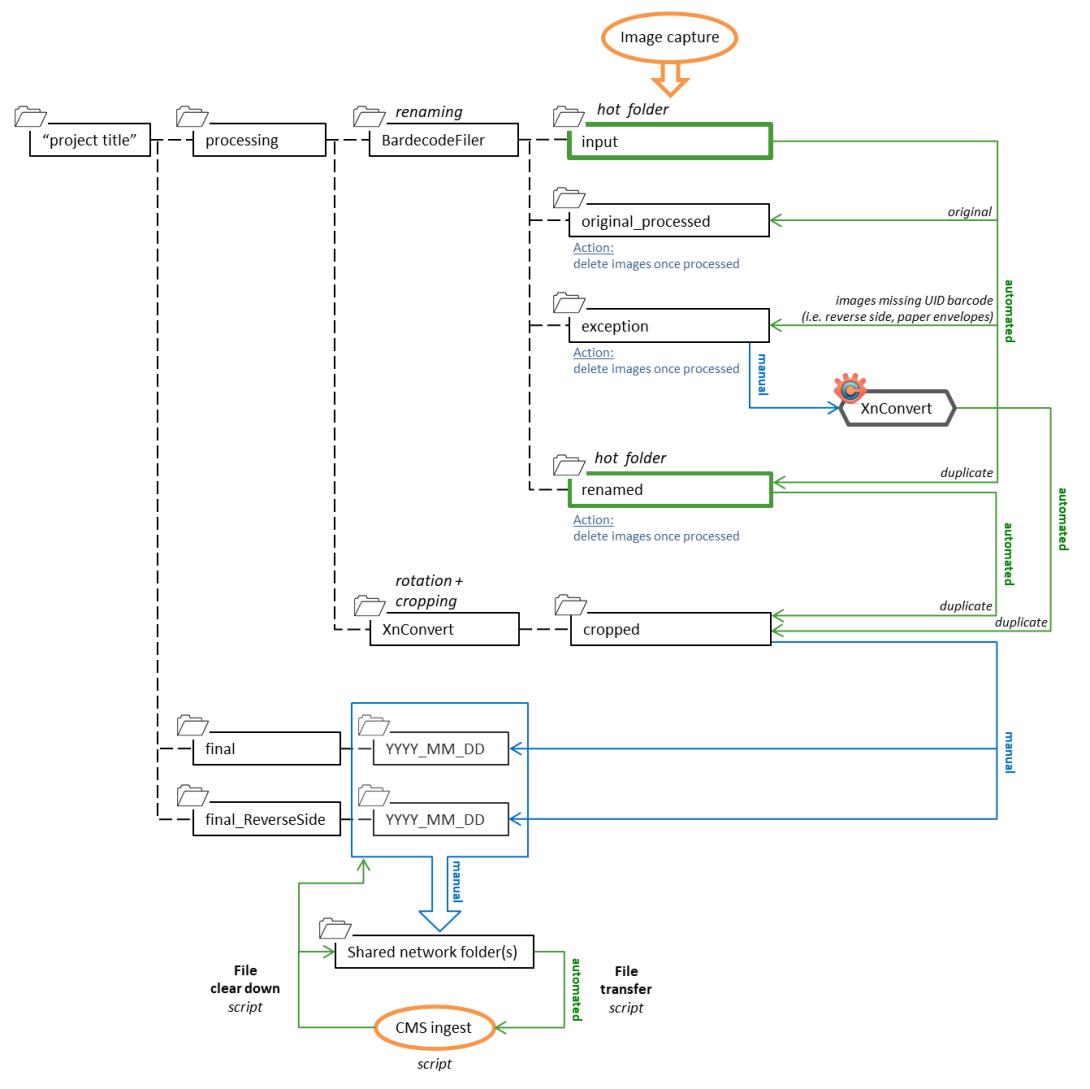
Folder structure and file processing using automated file renaming and image processing. Legend: Dashed lines - folder structure; green lines - automated steps; blue lines - manual steps; orange ovals - processes.

**Figure 7.**
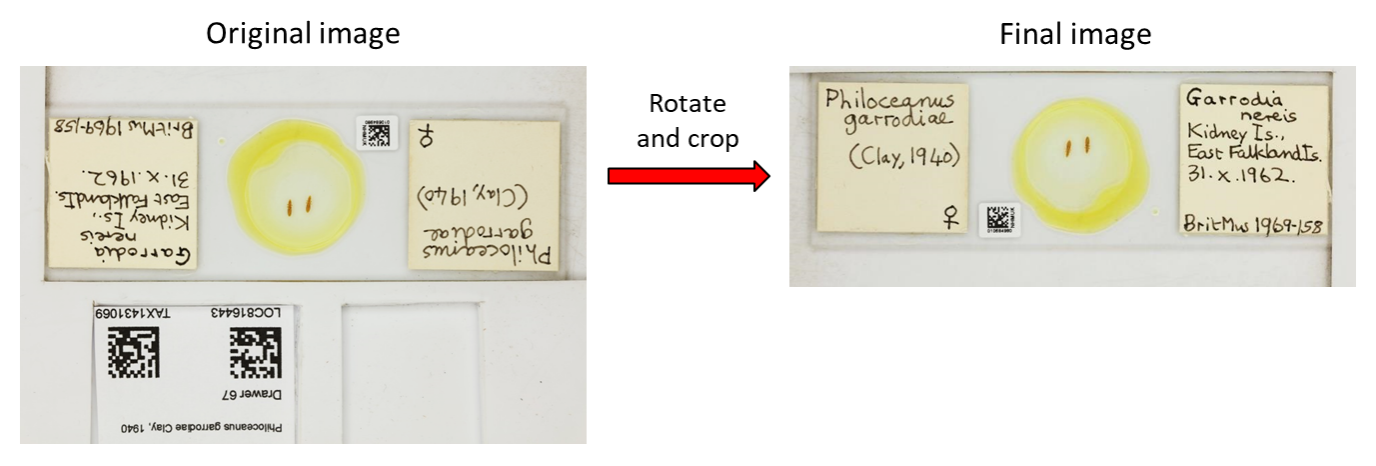
Example of the final specimen image, rotated and cropped using XnConvert, ready for ingestion into the collection management system.

1. Off-the-shelf software and hot folders were used for automated file renaming and image processing following image capture (Fig. 6). The software BardecodeFiler v.2.4.4.1 (http://www.bardecode.com/en1/app/bardecodefiler/) was set to watch the ‘input’ hot folder for new image files. Upon detection, these files were renamed and transferred to a second hot folder (‘renamed’) for automated image processing using XnConvert v.1.74 (https://www.xnview.com/en/xnconvert/) and saved to the ‘cropped’ folder.

- File renaming: the BardecodeFiler software was set to read the three barcodes in the image, UID catalogue number (attached to slide) and location and taxon primary key values (temporary label; Fig. 5b). This process was carried out using predefined rules in BardecodeFiler and the files were renamed as follows: “UIDBarcode_LocationPrimaryKey_TaxonPrimaryKey.jpg”.
- Image processing: XnConvert was set to rotate the images 180° and crop at specific coordinates to remove the temporary location and taxon primary key label from the final image (Fig. 7).
- Metadata: information associated with the image (owner’s name, author and copyright) was automatically written to the file EXIF data during image capture using EOS Utility.

2. If the UID barcode was missing in the image i.e. reverse image of a slide, paper envelope etc., the image file was saved to the ‘exceptions’ folder and renamed to “UIDBarcode_additional”.

- Renaming: the BardecodeFiler software uses the previously read UID barcode to rename the file.

3. At the end of each day, the image files in ‘cropped’ and ‘exceptions’ are manually quality checked.

4. The renamed and processed files in ‘cropped’ are then transferred to a date folder within ‘final’, ready to be copied to the staging area for ingestion into the Museum's CMS using the specimen record creation script. The image files in ‘original_processed’ and ‘renamed’ are then manually deleted.

5. Any additional images saved to ‘exceptions’ are manually copied to the XnConvert software to initiate the automated image processing (rotation and cropping). The images are saved to the ‘cropped’ folder and then transferred to a date folder within ‘final_ReverseSide’, ready to be copied to the staging area for ingestion into the CMS using the record attachment script. The image files in ‘exceptions’ are then manually deleted.

6. The image files in ‘final’ and ‘final_ReverseSide’ were copied daily to the staging area for ingestion into the CMS and then openly published through the Museum’s Data Portal (http://data.nhm.ac.uk, Shchedrina et al. 2017).

- Scripts were used to automate the file transfer within the staging area, as well as the clear down of the original image files from the imaging computer once these files had been ingested into the CMS.

## Discussion

To increase the efficiency and accuracy of digitisation workflows, we need to decrease the time taken for image capture and processing, simplify processes and reduce the opportunity for human error. The Phthiraptera slide digitisation project accomplished this by reducing the number of steps required to obtain a single renamed image of a slide; increasing the use of automated processes to rename and process image files; and adopting standardised pre-digitisation preparation of the collection. This adapted workflow allowed the entire microscope slide collection of parasitic lice (70,663 slides) to be digitised and made accessible via the Museum’s Data Portal in eight months using a single digitiser. This individual imaged and processed an average of 696 slides per day (circa 7 working hours), with a maximum real world rate of 1,006 slides per day for uncomplicated sections of the collection such as unidentified accessions, where the variation in specimen data was minimal (Table 1). The pre-digitisation preparation of the collection is not included in these daily rates as it is part of routine curation. When comparing the base rates achieved by trained digitisers during focused testing (i.e. only digitisation activities occurring), we were able to achieve substantially higher rates using the current automated workflow compared to the previous multi-slide and image segmentation approach (757 versus 613 slides per person per day; Table 1; Summerfield et al. 2019). In addition to higher digitisation rates, the proportion of errors was substantially reduced when using the automated workflow (current project: 0.006%, previous pilot: 0.09% for digitisers and 2.0 - 10.9% for volunteers). All of these errors were the result of slides that were missing a UID barcode, which was identified during the imaging process when the file was saved to the ‘exceptions’ folder. It is important to note, however, that the base rate for slide digitisation using our current automated workflow will vary between collection types depending on a number of factors. For example, slides housed in plastic / paper envelopes or stored vertically versus horizontally, will result in increased handling time, while slides that require multiple images i.e. double side slides, paper envelopes etc, will result in increased imaging time per slide.

In the Phthiraptera slide digitisation project, we imaged each slide individually rather than utilising the multi-slide and image segmentation approach used in our previous slide digitisation pilot (Summerfield et al. 2019). We initially thought that imaging multiple slides in a template would simplify and standardise the process, but in practice, this approach required a number of different steps and processes to create a single slide image, which added additional layers of complexity that were subject to potential failure and required regular troubleshooting. Even when automated processes were batch completed overnight, the complications of image processing across multiple stages, over several days, generated unnecessary complexity that slowed the overall process. By imaging slides individually, we were able to simplify the process, thus reducing the number of steps required to create a single slide image, which significantly reduced the imaging time per slide.

The streamlining of the image capture process into a single action also enabled us to replace the manual and semi-automated processes associated with the file renaming and post-processing with more automated (scripted) systems. The automated renaming replaced the use of *Inselect* to read the UID barcode on each segmented slide (a semi-automated step) and the manual annotation and verification of metadata, thus reducing the potential for human error associated with manual file renaming. This automated file renaming was made possible through pre-digitisation preparation of the collection and insertion of labels that encoded location and taxonomic metadata in barcodes. The use of a fixed imaging template ensured that the slides were consistently positioned in the same location, thus enabling automated image rotation and cropping. To significantly reduce post-processing time, multiple hot folders were used to enable file renaming and image processing to run in the background in real time, while the digitiser continued to image slides.

For the Phthiraptera slide digitisation project, location and taxonomic metadata were sufficient for inventory record creation; however, the workflow can be adapted to capture more data during imaging through the inclusion of additional temporary labels in the image. For example, type status or geographical region can be captured if collections are arranged as such. This multiple barcode digitisation workflow can also be adapted for use with other collection types such as pinned insects, herbarium sheets and spirit preserved material.

As with most workflows that focus on high-throughput digitisation and the production of digital collection inventories, it does not create images that are adequate for specimen-based research, although it does support subsequent label data capture and associated research. Digital inventories are also important for providing increased access to specimens and data, as well as enabling condition checking, which can be automated using computer vision approaches and prioritisation of specimens for subsequent high resolution and specialised imaging. As part of the Phthiraptera slide digitisation project, we imaged a representative of each species, focusing on type material where present, using a modified histology slide scanner, ZEISS Axio Scan.Z1. As expected, a number of slides were unsuitable for the slide scanner and, as a result, specimen imaging for these slides was carried out using a Canon EOS 5DS R with the MP-E 65mm lens, StackShot Macro Rail system (Cognisys Inc.) and a custom flashbox (Allan et al. 2018b). The custom DSLR-StackShot system provided similar resolution to that obtained using a 5x objective on the Axio Scan and was able to provide the flexibility needed to image these non-standard natural history slides.

In conclusion, this workflow demonstrates that pre-digitisation preparation, process simplification and careful use of automation, were more efficient and effective for digitisation of this large collection. In this particular case, direct use of primary keys from the Museum’s CMS avoided the bottleneck of data ingestion into the CMS, allowing the data to be rapidly accessed through our Data Portal.

## Figures and Tables

**Table 1.** Estimation of the base digitisation rate (slides per person per day) for the current Phthiraptera slide digitisation project versus the previous slide digitisation pilot.

	Slide digitisation pilot* (multi-slide and image segmentation)		**Phthiraptera slide digitisation project** (single slide and automated processing)	
	Volunteers (real world)	Digitisers (focused testing**)	Digitisers (focused testing**)	Digitisers (real world)
**Min** (a)	59	505	476	370
**Max** (b)	768	749	1,103	1,006
**Median** (m)	173	606	741	700
**Base Rate** *E* = (a+4m+b)/6	253	613	757	696
**Standard Deviation** SD = (b-a)/6	118	41	105	106
**Error Rate** (%)	2.0 - 10.9	0.09	0.006	

* values obtained from Summerfield et al. (2019)

** focused testing = only digitisation activities occurring
